# Crystal structures of Val58Ile tryptophan repressor in a domain-swapped array in the presence and absence of l-tryptophan

**DOI:** 10.1107/S2053230X21006142

**Published:** 2021-07-30

**Authors:** Janina Sprenger, Catherine L. Lawson, Claes von Wachenfeldt, Leila Lo Leggio, Jannette Carey

**Affiliations:** a Department of Chemistry, University of Copenhagen, DK-2100 Copenhagen, Denmark; b Center for Molecular Protein Science, Lund University, SE-221 00 Lund, Sweden; c Institute for Quantitative Biomedicine, Rutgers University, Piscataway, NJ 08854, USA; d Department of Biology, Lund University, SE-221 00 Lund, Sweden; e Chemistry Department, Princeton University, Princeton, NJ 08544, USA

**Keywords:** crystalline protein gel, hostal system, fragment-based screening, ligand binding, molecular baits, domain swapping, Val58Ile tryptophan repressor

## Abstract

The crystal structures of domain-swapped tryptophan repressor (TrpR) variant Val58Ile before and after soaking with the physiological ligand l-tryptophan (l-Trp) indicate that l-Trp occupies the same location in the domain-swapped form as in native dimeric TrpR and makes equivalent residue contacts. This result is unexpected because the ligand binding-site residues arise from three separate polypeptide chains in the domain-swapped form. This work represents the first published structure of a domain-swapped form of TrpR with l-Trp bound. The presented structures also show that the protein amino-terminus, whether or not it bears a disordered extension of about 20 residues, is accessible in the large solvent channels of the domain-swapped crystal form, as in the structures reported previously in this form for TrpR without N-terminal extensions. These findings inspire the exploration of l-Trp analogs and N-terminal modifications as labels to orient guest proteins that cannot otherwise be crystallized in the solvent channels of crystalline domain-swapped TrpR hosts for potential diffraction analysis.

## Abstract 2

In a domain-swapped, gel-like crystalline form of *Escherichia coli* tryptophan repressor, the physiological ligand l-tryptophan binds equivalently as in the native dimeric repressor, even though the binding-site residues originate from three distinct polypeptide chains instead of two, and large solvent channels accommodate a disordered N-terminal extension. Proteins that cannot otherwise be crystallized might be oriented in the channels for diffraction analysis by exploiting these features.

## 1. Introduction   

**Figure 1:**
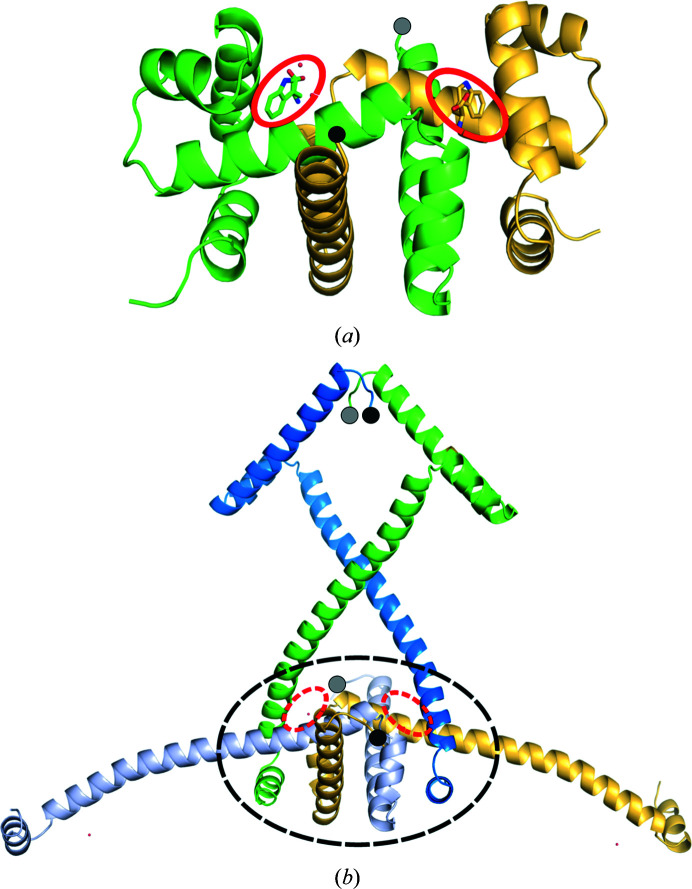
Dimeric and domain-swapped TrpR structures. (*a*) Dimeric Val58Ile TrpR (PDB entry 1jhg; Lawson, 1996*a* ▸) and (*b*) domain-swapped wild-type TrpR (PDB entry 1mi7; Lawson *et al.*, 2004 ▸) in schematic ribbon view. The four polypeptide chains comprising one dimer-like ‘node’ (black dashed oval) of the domain-swapped structure as described in the text are shown, each in a different color. l-Trp ligands in stick representation are enclosed in red ovals in the dimer; dashed red ovals mark equivalent positions in the domain-swapped structure, highlighting how each site in the domain-swapped node comprises residues arising from three chains, rather than from two as in the dimer. The N-termini of all chains are marked by black and gray filled circles, with black indicating a position in front of the plane of the page and gray a position behind the page.

Tryptophan repressor (TrpR) was one of the earliest gene-regulatory proteins for which a high-resolution crystal structure was determined in complex with its DNA regulatory site (Otwinowski *et al.*, 1988 ▸). The TrpR dimer (Fig. 1 ▸ *a*) is formed from deeply intertwined monomers (Schevitz *et al.*, 1985 ▸) and is related to domain-swapped proteins (Carey *et al.*, 2007 ▸), even though it does not meet a proposed definition predicated on the existence of a stably folded monomeric structure (Liu & Eisenberg, 2002 ▸), which TrpR apparently does not have (Shao *et al.*, 1997 ▸). Under certain conditions of crystal growth, TrpR dimers partially unfold and self-associate in a process akin to runaway domain swapping (Liu & Eisenberg, 2002 ▸), forming extended, higher-order domain-swapped structures connected by dimer-like nodes, each made up of four distinct polypeptide chains (Lawson *et al.*, 2004 ▸; Fig. 1 ▸ *b*). Formation of the extended domain-swapped structure depends on the conversion of two interhelical turns in each subunit of native dimeric TrpR into helical segments, resulting in the formation of a continuous, nearly straight α-helix of 47 residues. The resulting crystals of domain-swapped TrpR (ds-TrpR) have very large solvent channels and can be described as crystallinely ordered gels based on the chemical definition of gels, namely chain entanglement and high solvent content; IUPAC defines a gel as a nonfluid polymer network expanded throughout its whole volume by a fluid (Jones *et al.*, 2008 ▸). The highly unusual feature of ds-TrpR relevant to considering it as a crystalline gel is the extraordinary degree of chain entanglement that extends throughout the entire crystal, promoting a robust crystal lattice, as shown here, despite a very high solvent content that would ordinarily be associated with notably fragile protein crystals. To the best of the authors’ knowledge no other protein crystal qualifies as a crystallinely ordered gel, not even other domain-swapped examples, which have less extensive swapping of their parts and lower solvent contents. The ability of crystalline ds-TrpR to incorporate 5-bromotryptophan, a derivative of the physiological TrpR ligand l-tryptophan (l-Trp), has been reported previously (Lawson *et al.*, 2004 ▸), although the corresponding crystal structure was not deposited, nor has any structure been reported for ds-TrpR with l-Trp.

TrpR crystals in the typical native dimeric form have previously been reported for a variant in which Val58 is replaced by isoleucine (Val58Ile), one of 19 TrpR mutants created by Arvidson *et al.* (1991 ▸) to explore the role of the identity of the residue at position 58, which forms part of the l-Trp-binding site. Crystals of dimeric Val58Ile TrpR were used for high-resolution crystallographic and neutron diffraction analyses of l-tryptophan binding (Lawson, 1996*a* ▸; Daniels *et al.*, 2003 ▸) due to their facile growth to a very large size. The Val58Ile TrpR dimer structure (Lawson, 1996*a* ▸) is indistinguishable from that of the wild-type TrpR dimer (Lawson *et al.*, 1988 ▸), and the Val58Ile variant has wild-type TrpR activity *in vivo*, except for being slightly less sensitive to the l-Trp analog 5-methyltryptophan (Arvidson *et al.*, 1991 ▸, 1994 ▸). Structural analysis of Val58Ile TrpR suggested the basis for this alteration (Lawson, 1996*b* ▸), indicating that the l-Trp-binding site can accommodate an extra methyl group on the l-Trp ligand or an extra methylene group on the Val58 side chain, but that when these extra groups are present on both the protein and the ligand, the structural adjustments that would be required to accommodate them may account for the reduced affinity of 5-methyltryptophan for Val58Ile TrpR.

During the course of studies to pursue further analysis of Val58Ile TrpR by neutron diffraction based on promising early results (Lawson, 1996*a* ▸; Daniels *et al.*, 2003 ▸), it was discovered that this mutant could be crystallized in the extended domain-swapped form under the conditions reported previously for wild-type TrpR. The very large solvent channels typical of ds-TrpRs are being analyzed for possible use as a host system to facilitate diffraction analysis of guest proteins that cannot be crystallized (a so-called hostal system; Sprenger *et al.*, 2021 ▸). This system potentially offers a novel approach to the incorporation of biomolecular guests into porous systems, an area of general interest in applied biotechnology today (Yan *et al.*, 2015 ▸; Abe & Ueno, 2015 ▸; Hartje & Snow, 2018 ▸). Successful diffraction analysis of guest proteins in any host system requires uniform guest orientation to achieve crystallographic occupancy. In this regard, the ds-TrpR system offers a possible advantage if the guest can be targeted to the l-Trp-binding site using a label based on l-Trp. The present work was thus carried out using the abundantly available crystals of Val58Ile ds-TrpR soaked with l-Trp to evaluate the ability of ds-TrpR to bind l-Trp and to define the ligand orientation, which are here documented for the first time for any ds-TrpR. The results show that l-Trp binds in the same location and orientation as in typical dimeric TrpRs and makes equivalent residue contacts, affirming its potential as a label to potentially orient guests.

In the course of this work, crystals were also obtained and a structure was solved for Val58Ile ds-TrpR with a 23-residue N-terminal extension including a His_6_ purification segment, a spacer segment and a protease-cleavage sequence. Electron density for the N-terminal extension is visible in the solvent channel, but only two of its residues could be modeled due to disorder. The crystallizability of ds-TrpR with a flexible extension accessible in the solvent channels suggests the N-terminus as another locus for the attachment of groups that might orient guest proteins within the channels. All Val58Ile ds-TrpR structures solved here, with and without the N-terminal extension and with and without l-Trp, share essentially identical overall structures with wild-type ds-TrpR.

## 2. Materials and methods   

### 2.1. Protein expression and purification   

**Figure 2:**
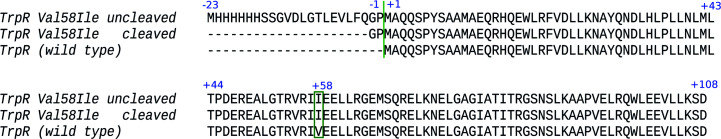
TrpR protein sequences. Protein sequences of the uncleaved TrpR Val58Ile variant including the N-terminal extension consisting of His_6_, spacer and protease-cleavage sequences (top), after cleavage of the extension (middle) and wild-type TrpR (bottom). The natural initiating methionine residue of wild-type TrpR is designated 1. Numbering of N-terminal extension residues begins with Pro -1 adjacent on the left to Met1, and continues with negative numbers to the new initiating methionine residue preceding the His_6_ sequence. Protease cleavage on the C-terminal side of Gln -3 yields the cleaved protein with additional N-terminal residues Gly-Pro preceding the natural methionine residue.

A DNA fragment encoding the Val58Ile TrpR variant (Fig. 2 ▸) with an N-terminal extension for subsequent purification containing a His_6_ segment (MHHHHHH) followed by a spacer (SSGVDLGT) and a rhinovirus 3C protease-cleavage sequence (LEVLFQ↓GP, with the arrow indicating the cleavage position; Cordingley *et al.*, 1989 ▸) was purchased from GenScript. The fragment was subcloned into plasmid pNIC28-Bsa4 (Savitsky *et al.*, 2010 ▸) and the resulting plasmid was transformed into *E. coli* T7 Express (New England Biolabs). The bacteria were grown in 1 l lysogeny broth (Difco) containing kanamycin at 50 µg ml^−1^ in 5 l baffled Erlenmeyer flasks at 37°C with agitation (200 rev min^−1^) until the cultures reached an optical density of 0.8 at 600 nm. Expression of Val58Ile TrpR was induced by the addition of isopropyl β-d-1-thiogalactopyranoside to 0.25 m*M* and growth of the culture was continued at 37°C for 4 h with shaking at 200 rev min^−1^. The cells were harvested by centrifugation at 8000*g* for 10 min at 4°C and the pellet was resuspended in ∼35 ml buffer *A* (50 m*M* sodium phosphate buffer with 300 m*M* NaCl, 20 m*M* imidazole pH 8.0) with the addition of one tablet of cOmplete EDTA-free protease inhibitor (Roche). The cells were broken by passage through a French press twice at 124 MPa; after centrifugation for 1 h at 100 000*g* at 4°C, the supernatant was filtered (Acrodisc syringe filter, 0.45 µm Supor membrane) and applied onto a HisTrap HP column (1 ml, GE Healthcare) pre-equilibrated with buffer *A*. Bound proteins were eluted with a linear gradient of 20–500 m*M* imidazole in buffer *A* over 20 column volumes. Fractions containing TrpR with the N-terminal extension were pooled and the concentration was determined using the absorbance at 280 nm with an extinction coefficient of 13 980 *M* ^−1^ cm^−1^ for TrpR with the extension. To remove the extension, the pooled fractions were mixed with His-tagged 3C protease at an enzyme:TrpR mass ratio of approximately 1:100, and the digestion mixture was transferred to a dialysis tube (32 mm, mass cutoff 3500 Da; Spectrapor) and dialyzed into buffer *A* containing 1 m*M* dithiothreitol (DTT) and 0.5 m*M* EDTA (as disodium salt) at 6°C for at least 12 h. After an additional dialysis step for 3 h at 6°C against 1 l buffer *A* without DTT and EDTA, the TrpR–protease mixture was applied as previously onto an equilibrated HisTrap HP column. The flowthrough containing cleaved TrpR was collected and dialyzed overnight against 1 l crystallization buffer (100 m*M* sodium phosphate buffer, 200 m*M* NaCl pH 7.5) containing 0.5 m*M* EDTA at 6°C and additionally against 1 l crystallization buffer for 12 h. Mass spectrometry confirmed that the N-terminus of the cleaved protein includes the expected N-terminal Gly-Pro residues (data not shown). The sequences of all proteins are shown in Fig. 2 ▸.

### 2.2. Crystallization   

#### 2.2.1. Val58Ile ds-TrpR with l-Trp   

Following the removal of the N-terminal extension by protease cleavage and before crystallization, purified Val58Ile TrpR was subjected to a final gel-filtration step using a Superdex 75 300/10 column with crystallization buffer. Pure fractions were pooled and concentrated to 3–8.5 mg ml^−1^ using 3000 Da Amicon Ultra-15 centrifugal filter units. Crystallization was performed using the hanging-drop vapor-diffusion technique according to previously reported conditions with a reservoir consisting of 27.5–35%(*v*/*v*) 2-propanol (iPrOH), 100 m*M* NaCl, 100 m*M* 2-[4-(2-hydroxyethyl)piperazin-1-yl]ethanesulfonic acid (HEPES) pH 7.5 (Lawson *et al.*, 2004 ▸). Crystals of Val58Ile TrpR grew within 1–2 days in drops consisting of 2–4 µl of a 1:1 mixture of protein and reservoir solutions. For X-ray diffraction, the crystals were soaked for 10 s in their reservoir solution to which ethylene glycol was added to 25%(*v*/*v*) as a cryoprotectant before cryocooling in liquid N_2_. To obtain the l-Trp-bound Val58Ile ds-TrpR structure, 1–2 day-old crystals were soaked for 30 min in cryosolution containing 10 m*M* l-Trp.

#### 2.2.2. Val58Ile ds-TrpR with the N-terminal extension   

Val58Ile ds-TrpR with the N-terminal extension (Fig. 2 ▸) was crystallized as described for the cleaved protein and purified identically except that proteolytic cleavage was not performed. No difference in protein purification nor in the growth or the behavior of the crystals was noted compared with the cleaved protein. Crystals of the uncleaved protein were harvested, cryocooled and measured as described for crystals of cleaved ds-TrpR.

### 2.3. X-ray data collection, processing and molecular replacement   

#### 2.3.1. Structure of Val58Ile ds-TrpR with and without l-Trp   

X-ray diffraction data from crystals of the cleaved TrpR Val58Ile variant (50–100 µm in the longest dimension) were collected on beamline ID30A-3 at ESRF and data from 10 m*M* l-Trp-soaked crystals were collected on beamline P11 at DESY, Hamburg. After cryoprotectant soaking and cryocooling as described above, 4000 frames were collected with 0.1° rotation, with a detector distance corresponding to about 0.5 Å below the diffraction limit of the crystals suggested by the initial processing. Processing was performed using *XDS* (Kabsch, 2010 ▸). The data sets of the best-diffracting crystals extended to 2.03 and 2.45 Å for Val58Ile ds-TrpR and l-Trp-soaked Val58Ile ds-TrpR crystals, respectively. Structures for these two single-crystal data sets were determined by molecular replacement using *MOLREP* (Vagin & Teplyakov, 2010 ▸) with the domain-swapped wild-type TrpR structure as a search model (PDB entry 1mi7; Lawson *et al.*, 2004 ▸). Alternating cycles of manual fitting and automated refinement including translation–libration–screw (TLS) refinement (for the l-Trp-bound structure) were carried out with *Coot* (Emsley *et al.*, 2010 ▸) and *phenix.refine* (Afonine *et al.*, 2012 ▸), respectively.

#### 2.3.2. Structure of Val58Ile ds-TrpR with the N-terminal extension   

Data were collected on beamline P13 at DESY, Hamburg. The crystals typically diffracted to 2.4–2.7 Å resolution, similar to the diffraction observed from crystals of cleaved ds-TrpR. The data sets after data reduction and processing with *autoPROC* (Vonrhein *et al.*, 2011 ▸) showed identical unit-cell parameters and space group to cleaved ds-TrpR. One of the best-quality data sets from the uncleaved ds-TrpR Val58Ile crystals was used for molecular replacement with the (cleaved) ds-TrpR Val58Ile structure (PDB entry 6st6) as a search model in *MOLREP* (Vagin & Teplyakov, 2010 ▸) and subsequent refinement with *REFMAC*5 (Murshudov *et al.*, 2011 ▸). Further manual and automated refinement was carried out as described for the cleaved structures above.

## 3. Results   

### 3.1. Crystal structure determination of Val58Ile ds-TrpR   

After protease cleavage, the Val58Ile TrpR protein contains an N-terminal Gly-Pro dipeptide preceding the original methionine residue, which in native wild-type TrpR is present as *N*-formylmethionine (fMet) and is largely processed off (Gunsalus & Yanofsky, 1980 ▸). Hereafter, the Val58Ile TrpR protein after protease cleavage is referred to as cleaved. Although the initiating fMet residue is mostly absent from native TrpR, by convention the numbering of the TrpR sequence begins with methionine as residue 1, and this convention is followed here for both the cleaved and uncleaved proteins for consistency with prior work. Additionally in this work, non-native residues prior to the original initiating methionine are given negative numbers beginning with -1 for the residue adjacent to the original methionine, as indicated in Fig. 2 ▸. The TrpR Val58Ile variant protein, including the N-terminal His_6_, spacer and protease cleavage sequences, contains 22 or 23 additional N-terminal residues depending on whether the initial fMet residue is processed off, which is unknown. The uncleaved protein is expected to include the initiating fMet residue, as inferred from mass spectrometry of similar N-terminally extended proteins (unpublished observations). This Val58Ile TrpR protein with intact N-terminal extension is hereafter referred to as uncleaved.

Val58Ile TrpR both with and without the N-terminal extension, *i.e.*, uncleaved and cleaved Val58Ile TrpRs, produced hexagonal bipyramidal crystals in 1–2 days under conditions identical to those that produced hexagonal bipyramidal crystals of wild-type TrpR in the domain-swapped form (Lawson *et al.*, 2004 ▸), and with no detected differences in the crystallization behavior between cleaved and uncleaved proteins or between Val58Ile and wild-type TrpR. Crystals of Val58Ile ds-TrpR varied in size from ∼50 to ∼300 µm in the largest dimension depending on the protein concentration used in crystallization (3–8 mg ml^−1^), with smaller crystals growing at higher protein concentrations. l-Trp at 10 m*M* final concentration in cryoprotectant solution was used to soak 1–2 day-old crystals for 30 min.

**Figure 3:**
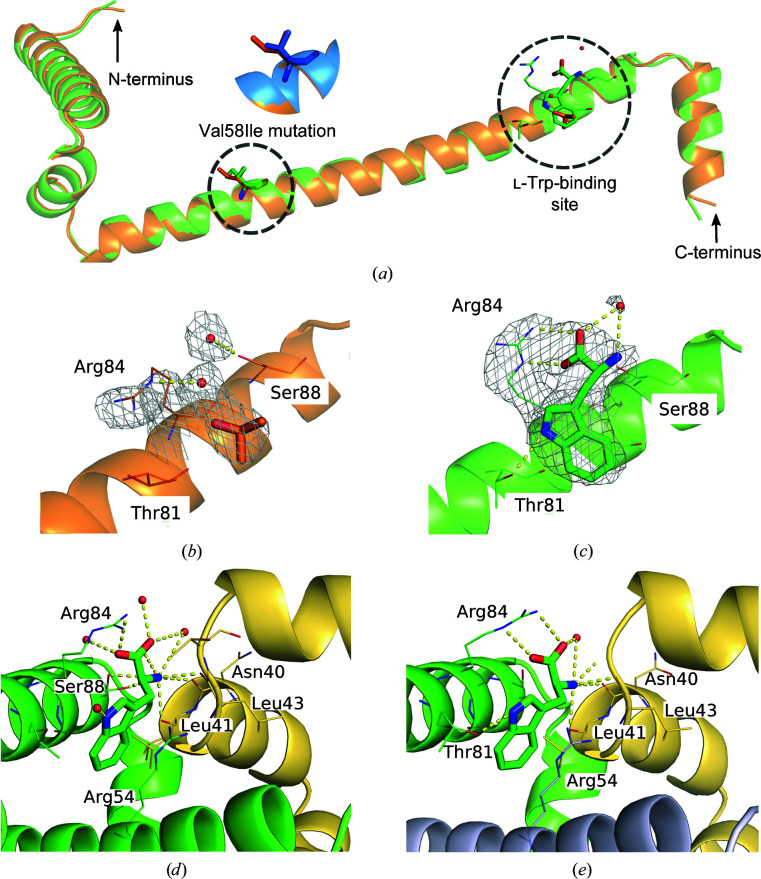
Structures of Val58Ile ds-TrpR without and with l-Trp. The polypeptide chain is represented as a ribbon cartoon in orange for the structure without l-Trp and in green for the structure with l-Trp. (*a*) Overlay of one polypeptide chain from each structure, with the N- and C-termini labeled. The location of the mutation giving rise to the Val58Ile variant is indicated by a dashed oval, with an enlargement above showing the Val58 and Ile58 side chains as sticks in an overlay of the local structures of wild-type ds-TrpR with Val58 (blue; PDB entry 1mi7; Lawson *et al.*, 2004 ▸) and of Val58Ile ds-TrpR with Ile58 (orange; this work). The l-Trp-binding region and its local surroundings including a water molecule (red oxygen sphere) are indicated by the dashed circle, where l-Trp is shown as sticks and the nearby residues Thr81, Arg84 and Ser88 (left to right) are shown as lines with atomic colors (oxygen, red; nitrogen, blue; carbon, same color as main chain). (*b*) The circled area in (*a*) is represented by a 2*F*_o_ − *F*_c_ electron-density map at contour level 1.0σ shown as a gray mesh surrounding Arg84 and the modeled ligands iPrOH (orange C atoms) and water (red oxygen sphere), with potential hydrogen-bond interactions with neighboring residues shown as dashed yellow lines. Only residues inferred to interact with the ligands are labeled. (*c*) As in (*b*) for the l-Trp-soaked structure. (*d*) Details of l-Trp binding, using the chain colors in (*a*), are shown for the structure of dimeric Val58Ile TrpR (PDB entry 1jhg; Lawson, 1996*a* ▸). (*e*) As in (*d*) for the structure of Val58Ile ds-TrpR (this work).

The structures of free and l-Trp-soaked cleaved Val58Ile ds-TrpR were determined to 2.05 and 2.45 Å resolution, respectively, and refined to an *R*_work_/*R*_free_ of 26.8/29.9% and 27.8/29.1%, respectively. The structures of cleaved Val58Ile ds-TrpR with and without l-Trp soaking have been deposited in the Protein Data Bank (PDB) as entries 6st6 and 6st7, respectively. Processing and refinement statistics are shown in Table 1 ▸, and Fig. 3 ▸(*a*) gives an overview of the structures and their prominent features. The structure of uncleaved TrpR is considered in a separate section below.

The final *R*_free_ values of cleaved Val58Ile ds-TrpR models both with and without l-Trp soaking are slightly above the median of 24–26% reported by Shao *et al.* (2017 ▸) in an analysis of recently determined crystal structures at similar resolution. These *R*_free_ values indicate that the structure models are basically correct; the slightly high *R*-factor values are likely to reflect difficulties in accurately modeling the extensive solvent regions (comprising 75% of the total crystal volume; Matthews coefficient *V*_M_ = 5.11 Å^3^ Da^−1^), as well as the complex solvent composition, which includes water [∼50%(*v*/*v*)], iPrOH [∼25%(*v*/*v*)] and ethylene glycol [∼25%(*v*/*v*)], any of which may be partially ordered at the protein–solvent interface but could not be modeled. As suggested in the original study of wild-type ds-TrpR (Lawson *et al.*, 2004 ▸), deviations from an ideal crystal lattice may reflect minor displacements along the highly extended polypeptide network. TLS refinement to account for positional displacements of atoms in the crystal was applied, and resulted in improved agreement with the diffraction data for the l-Trp-soaked crystals. Diffraction data from crystals with and without l-Trp had high Wilson *B* factors after processing (>50 Å^2^), and the average atomic *B* factors (>70 Å^2^) for the refined structures are higher than expected for their nominal resolutions.

The structures of cleaved Val58Ile ds-TrpR with and without soaked l-Trp have only minor differences in overall structure (r.m.s.d. of 0.56 Å for all non-H protein atoms). The most prominent deviations (0.7–1.2 Å) are at C^α^ positions close to the l-Trp site and in the C-terminal region (residues 75–102). The Val58Ile substitution has no effect on the overall domain-swapped structure relative to the wild-type ds-TrpR structure (Lawson *et al.*, 2004 ▸; PDB entry 1mi7), with an r.m.s.d. over all protein atoms of 0.36 Å. The structures reported here (Table 1 ▸) have improved geometry compared with the previously reported ds-TrpR structure but have slightly higher average *B* factors, whereas the *R* factors are in a similar range.

### 3.2. Interactions of the l-Trp ligand   

At the expected position of the l-Trp-binding site, the structure of cleaved Val58Ile ds-TrpR without l-Trp soaking shows electron density that could be modeled as one iPrOH and two water molecules positioned to interact with Arg84 and Ser88 (Fig. 3 ▸ *b*), similar to the previously reported ligand-free wild-type ds-TrpR (Lawson *et al.*, 2004 ▸). Although the density somewhat resembles l-Trp, comparison with the unambiguous electron density for l-Trp obtained after soaking (Fig. 3 ▸ *c*), and the orientations of the side chains implicated in l-Trp interactions, indicate that l-Trp is not present in the l-Trp-free form. In the l-Trp-soaked form distinct changes are observed in the side-chain conformations of Arg84, which comes within hydrogen-bonding distance of the l-Trp carboxylate, and of Thr81, which rotates to within hydrogen-bondimg distance of the indole ring N atom. The l-Trp ligand has an average *B* factor of ∼75 Å^2^, which is lower than the overall *B* factor of the structure (96 Å^2^; Table 1 ▸) and is in the range of the surrounding residues (∼70–90 Å^2^). The modeled iPrOH and two water molecules in the l-Trp site of the l-Trp-free structure have an average *B* factor of ∼65 Å^2^, which is slightly lower than the overall *B* factor of this structure (74 Å^2^; Table 1 ▸) and is in the range of the surrounding residues (∼40–70 Å^2^). The conclusion that l-Trp is absent from the ligand-binding site of the unsoaked ds-TrpR crystals is consistent with all prior evidence from both X-ray and NMR results on apo TrpR in both native dimeric and ds-TrpR forms (see, for example, Zhang *et al.*, 1987 ▸; Hyde *et al.*, 1991 ▸; Zhao *et al.*, 1993 ▸; Lawson *et al.*, 2004 ▸; Carey *et al.*, 2012 ▸; Harish *et al.*, 2017 ▸). l-Trp has never been observed in crystals or NMR structures of TrpR purified as the apoprotein, and assays of purified apo TrpR always confirm its characteristic DNA-binding affinity (Carey, 1988 ▸), affirming the absence of l-Trp.

Each l-Trp ligand in l-Trp-soaked cleaved Val58Ile ds-TrpR crystals has a position and surrounding environment equivalent to each of the two l-Trp ligands in symmetric, dimeric Val58Ile TrpR (PDB entry 1jhg; Lawson, 1996*a* ▸; Fig. 3 ▸ *d*), which in turn are identical to the position and environment of the l-Trp ligands in wild-type dimeric TrpR structures (PDB entries 1wrp and 2oz9; Lawson *et al.*, 1988 ▸). In the dimeric structures each of the two equivalent l-Trp-binding sites is located at the interface between the two symmetrically intertwined chains, both of which contribute interactions to each ligand. From one chain the Arg84 guanidino group is positioned to interact with the l-Trp carboxylate, and the Thr81 hydroxyl group is positioned to interact with the l-Trp indole ring N atom; Ser88 has been reported to adopt different conformations and can be a direct or water-mediated hydrogen-bonding partner for the l-Trp α-amino group, and the Arg54 methylene groups pack against one ‘face’ of the l-Trp indole ring. In the second chain of dimeric TrpR the backbone carbonyl O atoms of Leu41 and Leu43 are positioned to interact with the l-Trp α-amino group, with Asn40 as an additional potential hydrogen-bonding partner. All of these potential ligand interactions are maintained in Val58Ile ds-TrpR, except that the origin of Arg54 is a third protein chain rather than the first chain as in dimeric TrpR (Fig. 3 ▸ *e*). In the domain-swapped form the long extended helix positions Arg54 away from the ligand-binding site formed by residues of its own chain, bringing it instead into an equivalent position at the ligand-binding site of the next dimer-like node of the extended array. Thus, in Val58Ile ds-TrpR the physiological ligand l-Trp occupies the equivalent binding site and engages in all of the same functional-group interactions as in native dimeric TrpR. This first reported structure of l-Trp bound to any domain-swapped TrpR reveals the remarkable finding that l-Trp binds identically in dimeric and ds-TrpRs despite the differences in the chain origins of the residues in their respective ligand-binding sites.

**Figure 4:**
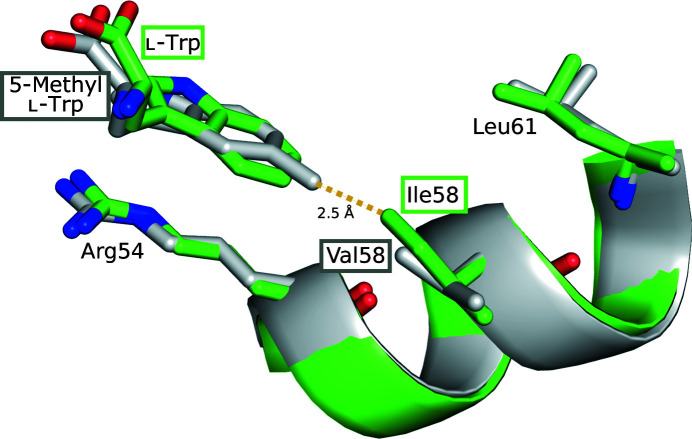
Overlay of part of the ligand-binding sites of the Val58Ile ds-TrpR structure with bound l-Trp (green boxes around the labels for Ile58 and l-­Trp) and the dimeric structure of wild-type TrpR with bound 5-methyl l-­Trp (PDB entry 6f9k; gray boxes around the labels for Ile58 and 5-MT). For simplicity only a short helical segment (cartoon ribbon) of each TrpR structure was overlaid in an orientation similar to that of Fig. 1 ▸(*a*). Ligands and nearby residues are shown as stick models with red O atoms and blue N atoms. The dotted yellow line indicates the distance between the Ile58 methyl group of Val58Ile ds-TrpR and the ligand 5-methyl group from the overlaid structure of wild-type TrpR with bound 5-MT.

Given the long-term goal of using ds-TrpRs as crystalline hosts for proteins that may be modified with l-Trp-based labels to orient them in the ligand-binding site, it was of interest to evaluate how the binding site compares in its tolerance towards modified ligands. To this end, a comparison of binding sites was made between Val58Ile ds-TrpR with bound l-Trp and dimeric TrpR with bound 5-methyl l-Trp (5-MT; PDB entry 6f9k). This choice was influenced by the previous proposal, based on the dimeric structure of Val58Ile TrpR (PDB entry 1jhg; Lawson, 1996*a* ▸), that dimeric TrpR can accommodate an extra methyl group on either the protein or the ligand but not both. The two structures were aligned by overlaying their local helical segments around residue 58, *i.e.*, Arg54–Leu61. Fig. 4 ▸ shows that the local alignment is excellent, including the side chains of residues Arg54 and Leu61. The superposition shows that as in dimeric TrpR, Val58Ile ds-TrpR presents a steric clash with bound 5-MT, with a predicted closest approach distance of 2.5 Å.

### 3.3. Structure of uncleaved Val58Ile ds-TrpR   

**Figure 5:**
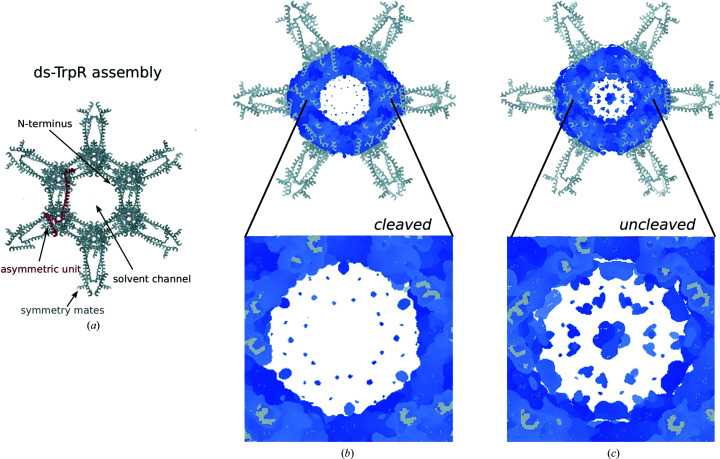
Electron density in the solvent channels of cleaved and uncleaved ds-TrpR. An assembly of the cleaved Val58Ile ds-TrpR crystal structure is shown in (*a*) that was made in *Coot* (Emsley *et al.*, 2010 ▸) by expanding the single TrpR molecule in the asymmetric unit (red ribbons) with symmetry mates (gray ribbons) to cover the crystal solvent channel. Structural assemblies as in (*a*) are shown for cleaved and uncleaved Val58Ile ds-TrpR in the upper panels in (*b*) and (*c*), respectively. Electron density is presented as a 2*F*_o_ − *F*_c_ map (blue) with 0.45 *I*/σ and map blurring with *B* ^2^ = 133 Å^2^. The lower panels in (*b*) and (*c*) present magnified views of the channel area of the assemblies shown in the top panels. Relative to (*a*), the assemblies in (*b*) and (*c*) are shown rotated by 30° about the crystal 6_1_ screw axis to more clearly show features of interest.

The structure of uncleaved Val58Ile ds-TrpR solved at 2.45 Å resolution (Table 1 ▸) shows electron density equivalent to residues -2 through 106 (22–131 in the numbering of the deposited structure) that could be assigned as in the cleaved protein. Additional electron density observed in the channels could be modeled by only two residues of the N-terminal extension (Gly -2 and Pro -1; Fig. 2 ▸). Both the l-Trp-soaked and unsoaked cleaved Val58Ile TrpR proteins contain these two residues after cleavage, but they are not visible in the electron density, with Met1 as the first residue that can be modeled in both cases. In the wild-type ds-TrpR structure previously deposited by Lawson *et al.* (2004 ▸) the first modeled residue is Ala2, although the protein contains fMet as the first N-terminal residue due to its incomplete removal *in vivo* from the overproduced protein (Gunsalus & Yanofsky, 1980 ▸). Connected but weak electron density that is unique to the uncleaved protein is detected in the solvent channels originating around Gly -2 (Fig. 5 ▸), consistent with the presence of the extension in the channel; this density is absent from the corresponding structure of cleaved ds-TrpR. Thus, whether or not the N-terminal extension is present, the N-terminus points into the channel (Fig. 5 ▸), as was observed previously for wild-type ds-TrpR crystals (Lawson *et al.*, 2004 ▸; Sprenger *et al.*, 2021 ▸). The remainder of the uncleaved Val58Ile ds-TrpR protein structure is essentially identical to that of cleaved Val58Ile ds-TrpR, except for the additional two N-terminal residues. The structure of uncleaved Val58Ile ds-TrpR is not further considered here except to note the relevance of the N-terminus as a modification site for potentially orienting guest proteins, as discussed below.

## 4. Discussion   

Substitution of isoleucine for Val58 does not alter the DNA- or l-Trp-binding activities of dimeric TrpR *in vivo* or *in vitro* (Arvidson *et al.*, 1991 ▸, 1994 ▸), and its overall dimeric structure is the same as that of wild-type TrpR (Lawson, 1996*a* ▸). The results presented here demonstrate that the structure of domain-swapped Val58Ile TrpR is also essentially identical to that of wild-type ds-TrpR. The results further indicate that the physiological ligand of dimeric TrpR, l-Trp, can occupy the expected ligand-binding site of Val58Ile ds-TrpR even though domain swapping replaces the native, intra-dimeric binding interface of dimeric TrpR with a binding interface created by three protein chains. Because the manner of l-Trp binding is otherwise indistinguishable from that of wild-type dimeric TrpR, and because Val58Ile ds-TrpR crystals are also indistinguishable from those of wild-type ds-TrpR, it is expected that l-Trp also binds in this manner to wild-type ds-TrpR. This conclusion is consistent with the previous report of 5-bromotryptophan incorporation into wild-type ds-TrpR crystals by soaking (Lawson *et al.*, 2004 ▸), with the Br atom localized in the l-Trp-binding site.

**Figure 6:**
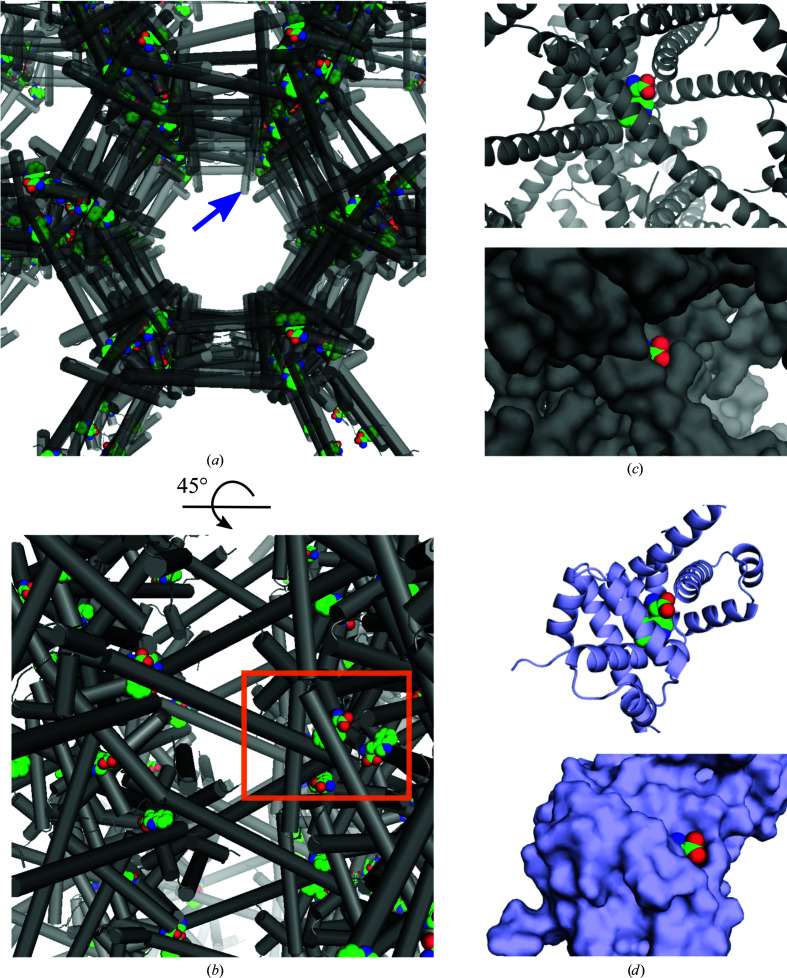
Solvent channels in Val58Ile ds-TrpR crystals. (*a*) View down the long solvent channel. The protein structure was expanded by *P*6_1_22 symmetry to show the continuous channel through the crystal. Protein helices are represented as gray cylinders. The position of one free N-terminus is marked by a blue arrow at the first ordered residue of the cleaved protein: Ala2 of the native TrpR sequence. l-Trp ligands are shown as CPK spheres in atomic colors with green C atoms. (*b*) Similar representation as in (*a*) but with the view rotated ∼45° along the indicated axis. The orange box encloses the area that is enlarged in (*c*). (*c*) View of the environment of one l-Trp ligand in Val58Ile ds-TrpR. The symmetry-related l-Trp molecules visible in (*b*) were deleted for clarity and to match (*d*). Protein helices are represented as gray ribbons (top) and as solvent-accessible surfaces (bottom). (*d*) A view equivalent to that in (*c*) for wild-type dimeric TrpR (PDB entry 1jhg; Lawson, 1996*a* ▸). Note the solvent-accessibility of the l-Trp α-carboxylate functional group.

Like wild-type ds-TrpR crystals, Val58Ile ds-TrpR crystals present solvent channels that form continuous, largely straight, pores throughout the crystal of ∼5 nm in diameter (Fig. 6 ▸ *a*). These wide channels presumably facilitate diffusion of the ligands l-Trp or 5-bromotryptophan into the crystals to reach the binding site. The channels are also large enough to admit small proteins (horse heart cytochrome *c* and human calmodulin; Sprenger *et al.*, 2021 ▸). The latter property has inspired the investigation of ds-TrpR as a crystalline host to enable the determination of structures of guest proteins that cannot themselves be crystallized, although to date the trial guest proteins cytochrome *c* and calmodulin have not been sufficiently ordered within the channels for structure solution. The availability of an apparently functional binding site for the native l-Trp ligand in Val58Ile ds-TrpR crystals, as shown in this work (Figs. 6 ▸ *a*–6 ▸ *c*) and inferred here for wild-type ds-TrpR, may offer the possibility of designing l-Trp-based or indole-based labels on guests that could lead to improved guest ordering in the channels of ds-TrpRs.

Many l-Trp analogs have been reported to bind to wild-type dimeric TrpR with affinities within approximately tenfold of that of l-Trp (Marmorstein *et al.*, 1987 ▸). Many of those analogs present modifications of the α-substituents of l-Trp (3-β-indoleacrylic acid, indole-3-butyric acid, indole-3-acetic acid, indole-3-propionic acid, tryptaphol, tryptamine, indole, *N*-formyl-l-tryptophan, indoline, l-abrine, 5-methyltryptamine, l-tryptophan hydroxamate, l-indole-3-lactic acid, l-tryptophan methyl ester and l-tryptophanamide), suggesting that this location is suitable for derivatization by a linker moiety for connection to a guest protein. The solvent-accessibility of l-Trp in Val58Ile ds-TrpR is essentially equivalent to that in dimeric wild-type TrpR (Figs. 6 ▸ *c* and 6 ▸ *d*), suggesting that chemically modified functional groups of l-Trp are likely to be tolerated similarly in dimeric TrpR and ds-TrpR. In addition, the N-terminus of wild-type (Lawson *et al.*, 2004 ▸) and Val58Ile ds-TrpR (Fig. 6 ▸ *a*) points towards the solvent channels and thus presents an alternative site that may be more tolerant of and/or solvent-accessible for modifications to anchor guest proteins. Indeed, the presence of the apparently disordered N-terminal extension does not reduce the resolution of the overall structure compared with the extension-free structure. The fact that the crystallization of TrpR in a domain-swapped form tolerates the presence of a disordered extension of over 20 residues encourages further work to explore a range of N-terminal extensions to use as bait for guest proteins or as a site for fusion of peptides or proteins that cannot otherwise be crystallized. These and other possibilities for using ds-TrpR as a crystalline host system have been discussed in more detail by Sprenger *et al.* (2021 ▸) and are being explored in ongoing work.

The evident similarity of l-Trp binding to dimeric TrpR and ds-TrpR suggests the possibility of employing dimeric TrpR in large-scale screening to identify l-Trp or indole-based analogs or even unrelated compounds that may be developed into potential labels that can be covalently attached to a guest protein and serve to orient guest proteins in ds-TrpR host crystals by binding at the l-Trp site. Screening of potential binding candidates in solution is not applicable to ds-TrpR because this form of the protein is presumably only highly populated in crystals. Fragment-based screening approaches such as those used in the identification of candidate drug leads (Shuker *et al.*, 1996 ▸) typically employ high-throughput analysis of low-molecular-weight compounds (the fragments) in large libraries to identify those with weak binding activity. The fragment method is predicated on the expectation that the chemical coupling of two or more weakly binding fragments improves affinity through non-additive entropic effects (Green, 1966 ▸; Tsallis, 2009 ▸; Bronowska, 2011 ▸). Success in a broad fragment-screening approach using dimeric TrpR appears to have the potential to greatly expand the range of chemical functionalities suitable as labels for guest proteins entrained in ds-TrpR host crystals.

In recent years, high-throughput strategies have also been adapted for fragment screening with crystals, primarily in the context of drug discovery or inhibitor design (Chilingaryan *et al.*, 2012 ▸; Patel *et al.*, 2014 ▸), enabling direct application to ds-TrpR. Individual compounds from fragment libraries are soaked into crystals before diffraction, and data analysis is performed with efficient pipelines including *XchemExplorer* (Krojer *et al.*, 2017 ▸), with identification of hits using *PanDDA* (Pearce *et al.*, 2017 ▸). These methods can allow screening on the scale of a few hundred candidates. One advantage of the crystal-screening method is that the pose of the soaked compound is resolved, which in the case of ds-TrpR will be important for predicting the disposition of a prospective ligand-labeled guest protein in the channels. This advantage will be critical for the TrpR system in particular because of the surprising finding (Lawson & Sigler, 1988 ▸) that indole­propionic acid (IPA) co-crystallizes with dimeric TrpR in the same binding site as l-Trp but in a ∼180° flipped orientation, with the indole ring N atom taking the place of the missing α-amino group. The affinity of dimeric TrpR for IPA determined by equilibrium dialysis in solution is reported to be *K*_d_ = 9.74 µ*M*, compared with 14.6 µ*M* for l-Trp (Marmorstein *et al.*, 1987 ▸). Given the high resolution of the IPA cocrystals (1.65 Å), this unexpected pose for a ligand whose structure and affinity are highly similar to those of l-Trp raises fundamental questions about the relationship between crystallo­graphic occupancy and ligand binding, with the following practical implications that must be confronted for success in a hostal-type approach, and which are of general relevance for crystallography as well.

Although ligand-binding affinity confirmed in solution implies that crystallographic occupancy may be achievable, the reverse is not necessarily true: the observation of a localized ligand in the phase-separated crystal system does not necessarily predict ligand affinity in solution at equilibrium. In the crystal lattice a ligand (from cocrystallization or soaking) will presumably orient itself to optimize any noncovalent bonding interactions that are possible in its local environment. Thus, localization to a crystallographic ‘binding’ site can occur even if that site is only marginally favored over other sites and binding is immeasurably weak in solution. A clear example of this effect is illustrated by the common observation of components of crystallization solvents in seemingly well localized sites on protein surfaces, as for iPrOH in the l-Trp site of the l-Trp-free ds-TrpR structure here, which has a *B* factor similar to those of its surrounding residues, as does l-Trp in the l-Trp-soaked structure. Binding affinity is a continuous quantity that has no theoretical lower limit among potential ligands of a given target, only the practical limit of detectability. Solvents and solutes can credibly be considered to be ligands whose affinities under equilibrium conditions are immeasurably small, even if their pose and interactions with the target are resolved crystallographically. The overall conclusion is that measurable affinity in solution cannot necessarily be inferred from crystallographic observation of a well ordered ligand. Thus, the ability to use the TrpR dimer for screening of ligand affinity in solution, and ds-TrpR to screen for ligand pose in crystals, will be important complementary approaches to identify candidate labels for a hostal-based structure determination.

Finally, the finding that the Val58Ile variant supports the formation of crystals in the domain-swapped array and has an apparently functional binding site for l-Trp suggests that other TrpR sequence variants may also be useful as crystalline hosts. Domain-swapped TrpR was first identified in a temperature-sensitive mutant, Leu75Phe (Lawson *et al.*, 2004 ▸), that also alters the l-Trp-binding site, although its crystal structure and its ability to bind l-Trp were not examined in the domain-swapped form. Altering the TrpR binding site thus offers another degree of freedom for designing potential labels to accommodate non-l-Trp-based ligands.

## Supplementary Material

PDB reference: domain-swapped tryptophan repressor, Val58Ile variant, 6st6

PDB reference: bound to l-tryptophan, 6st7

PDB reference: with N-terminal extension, 7os9

## Figures and Tables

**Table 1:** Crystallographic data for Val58Ile ds-TrpR with and without L-Trp Values in parentheses are for the highest resolution shell.

	Val58Ile TrpR (cleaved)	Val58Ile TrpR with L-Trp (cleaved)	Val58Ile TrpR (uncleaved)
PDB code	6st6	6st7	7os9
Data-collection and processing statistics			
Diffraction source	ID30A-3, ESRF	P11, DESY	P13, DESY
Resolution range (Å)	45.45–2.05 (2.12–2.05)	45.57–2.45 (2.54–2.45)	62.47–2.45 (2.54–2.45)
Wavelength (Å)	0.968	1.033	0.97625
Space group	*P*6_1_22	*P*6_1_22	*P*6_1_22
*a*, *b*, *c* (Å)	85.26, 85.26, 115.32	86.83, 86.83, 114.56	85.75, 85.75, 115.56
α, β, γ (°)	90, 90, 120	90, 90, 120	90, 90, 120
Total No. of reflections	713714 (73803)	103747 (10150)	361387 (41671)
No. of unique reflections	16145 (1570)	9881 (1543)	9744 (937)
Completeness (%)	99.88 (99.94)	99.9 (99.90)	99.95 (100.00)
Multiplicity	44.2 (47.0)	10.5 (10.6)	37.1 (44.5)
〈*I*/σ(*I*)〉	34.59 (1.74)	17.52 (0.78)	33.33 (2.78)
Wilson *B* factor (Å^2^)	51.11	81.03	93.79
*R*_merge_ (%)	7.6 (262.3)	6.27 (209.5)	7.0 (195.0)
*R*_meas_ (%)	7.7 (265.2)	6.582 (219.8)	7.2 (197.0)
CC_1/2_	1 (0.81)	1 (0.43)	0.99 (0.93)
CC*	1 (0.95)	1 (0.78)	1 (0.98)
Refinement statistics			
Final *R*_work_ (%)	26.76 (37.30)	25.53 (38.79)	25.46 (39.55)
Final *R*_free_ (%)	30.14 (35.27)	28.56 (50.83)	27.94 (42.32)
Clashscore	1.15	1.14	0.55
R.m.s. deviations			
Bonds (Å)	0.001	0.002	0.002
Angles (°)	0.38	0.42	0.41
Ramachandran favored (%)†	99.03	100	99.08
Ramachandran allowed (%)	0.97	0	0.92
No. of macromolecules	1	1	1
No. of protein residues	105	105	108
No. of ligands			
IPA	6	1	0
L-Trp	0	1	0
No. of waters	52	20	11
Average *B* factor (Å^2^)	73.88	96.33	113.92
No. of TLS groups	1	3	1

† According to analysis with *MolProbity* (Chen *et al.*, 2010 ▸).
